# TM-doping modulated p–d orbital coupling to enhance the oxygen evolution performance of Ni_3_S_2_†

**DOI:** 10.1039/d4na00503a

**Published:** 2024-08-16

**Authors:** Qiuhong Li, Minghao Zhang, Rui Wang, Jing Pan, Huailiang Fu

**Affiliations:** a School of Physics and Technology, Nantong University Nantong 226019 China fu.hl@ntu.edu.cn; b College of Physics Science and Technology, Yangzhou University Yangzhou 225002 China jp@yzu.edu.cn

## Abstract

The design of an ideal catalyst for the oxygen evolution reaction (OER) is essential for electrocatalytic water-splitting. The Ni_3_S_2_ (101) facet is considered a suitable electrocatalyst owing to its good conductivity and stability, but high performance remains a challenge. Our first-principles calculations show that transition metal (TM) doping can effectively modulate p–d orbital coupling resulting from TM doping-induced charge redistribution on active site Ni atoms, thus enhancing the orbital interaction between Ni-3d_*xz*_ and O-2p_*y*_ as well as between Ni-3d_*z*2_ and O-2p_*x*_. This improves the binding of the active site and oxygen-containing intermediates, thereby reducing the overpotential of the OER. Mo-doped Ni_3_S_2_ can be considered a compelling OER catalyst for its better stability and lower overpotential of 0.23 V.

## Abstract 2

TM doping modulates p–d orbital coupling to improve the OER performance of the Ni_3_S_2_ (101) facet.

## Graphical abstract

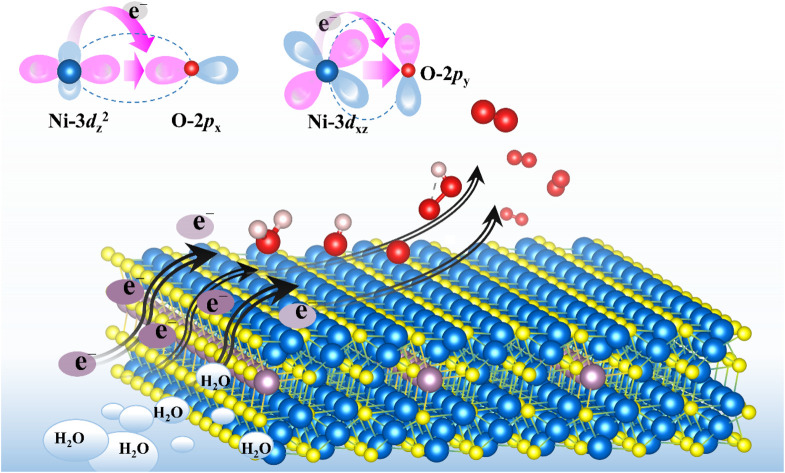

## 1. Introduction

Electrochemical water-splitting is a primary method for industrial hydrogen production and involves two half-reactions: the hydrogen evolution reaction (HER) and oxygen evolution reaction (OER).^1–4^ The HER involves relatively simple two-electron transfer steps,^5^ whereas the OER involves more complex four-electron transfer steps requiring a higher overpotential to overcome the reaction barrier,^6^ which determines the efficiency of water splitting.^7–10^ Noble metal oxides, such as RuO_2_ and IrO_2_,^11,12^ are considered ideal for OER electrocatalysts, and their OER properties can be further enhanced *via* metal doping. Chen *et al.* reported that Mn-doped RuO_2_ displayed outstanding OER activity with an overpotential of 158 mV at 10 mA cm^−2^ and 5000 cycle stability (0.5 mol L^−1^) in the presence of sulfuric acid.^13^ Hang *et al.* found that IrO_2_ doped with Cr or Mn exhibited high OER activity with a low overpotential of *η* = 275–230 mV at 10 mA cm^−2^ utilizing scanning transmission X-ray microscopy and high-resolution light emission spectroscopy analysis.^14^ Compared to noble metal oxides, transition-metal dichalcogenide Ni_3_S_2_ has attracted wide attention owing to its natural abundance, intrinsic metallic characteristics with distinctive conductivity, and better OER catalytic performance.^15–19^ To further enhance its catalytic activity, we doped the Ni_3_S_2_ (101) facet with transition-metal atoms (TM = Cr, Mn, Fe, Co, Cu, Zn, Mo, Ru, and Rh). Our first-principles calculations showed that the introduction of dopant TM atoms induced charge redistribution of active sites and strengthened the interaction between Ni-3d_*xz*_ and O-2p_*y*_ orbitals as well as between Ni-3d_*z*2_ and O-2p_*x*_ orbitals, thereby enhancing the interaction between the active site and oxygen-containing intermediates. As a result, TM doping improved the OER performance of the Ni_3_S_2_ (101) facet; among them, Mo doping had the best regulating effect on the OER process.

## 2. Computational model and methods

**Fig. 1:**
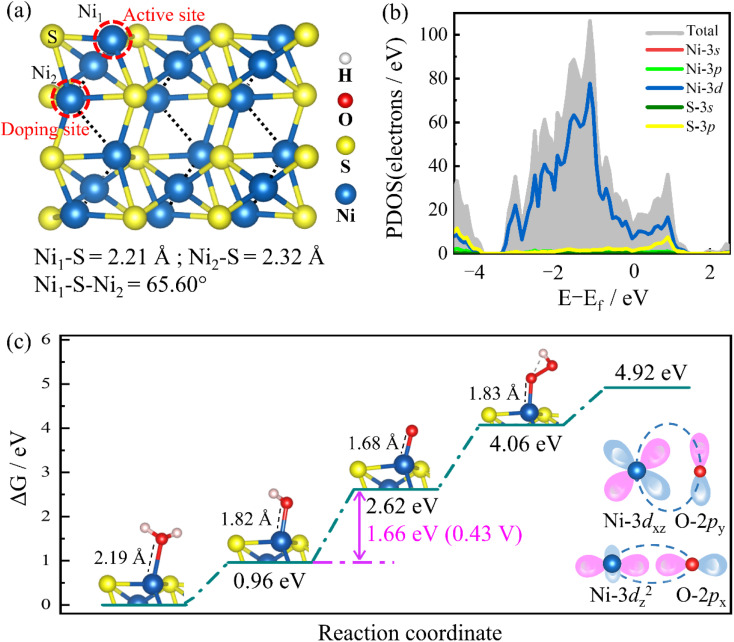
(a) Ni_3_S_2_ (101) facet model diagram, (b) atom-partial DOS and (c) free energy of the four steps during the OER process. The inset describes Ni-3d and O-2p orbital interactions.

Based on density functional theory (DFT), we used the Perdew–Burke–Ernzerhof (PBE) exchange correlation functional for generalized gradient approximation in the Vienna *ab initio* simulation package (VASP).^20–23^ The plane wave basis set had a cutoff energy of 450 eV, and electron–ion interactions were described using projection-enhanced plane waves. Monkhorst–Pack *k*-point grids of 7 × 7 × 1 and 11 × 11 × 1 were employed for geometric optimization and electronic construction. The convergence thresholds in energy and force were 1.0 × 10^−5^ eV and 0.01 eV Å^−1^, respectively. The free energy of the OER process was defined by the equation Δ*G* = Δ*E* + ΔZPE − *T*Δ*S*, where Δ*E*, ΔZPE, and Δ*S* represent the changes in energy, zero-point energy, and entropy contribution of the geometry, respectively, which were obtained by calculating vibrational frequencies of reactants and products in the gas phase using standard tables;^24^ the calculation details can be found in the ESI.† The Ni_3_S_2_ (101) facet was achieved experimentally;^15^ here, it was separated from the bulk Ni_3_S_2_ by a 15 Å vacuum layer. A 1 × 1 supercell with six layers of slabs was used, revealing a corrugated structure with three exposed Ni atoms and three S atoms on the surface. The bottom four layers were fixed, and the top bilayer was completely fully relaxed. TM atoms (Cr, Mn, Fe, Co, Cu, Zn, Mo, Ru, and Rh) were introduced to replace the Ni atom on the subsurface near the active site, as shown in Fig. 1(a), which is the optimum site for the dopant because TM doped Ni_3_S_2_ systems have good stability and catalytic activity in this case.^25^ Our previous work has shown that the GGA method is reliable with the calculated lattice parameters of *a* = *b* = 5.754 Å, and *c* = 7.063 Å,^25^ well agreeing with the experimental value (*a* = *b* = 5.745 Å, *c* = 7.063 Å).^26^ In ref. 25, we compare the GGA and GGA+U methods, and the results show that the band structure and free energy diagrams calculated using these two methods are almost the same. Additionally, we consider the relativistic and dispersion effects in the ESI;† the compared results show that the relativistic and dispersion effects have little influence on the OER process.

## 3. Results and discussion

### 3.1 Electronic structure of the TM-doped Ni_3_S_2_ (101) facet

As shown in Fig. 1(a), surface Ni_1_ is the catalytic active site determined by repeated testing. Many possible doping sites for TM atoms are considered. The optimum doping site is the Ni_2_ site because this doping site has good stability and has a positive effect on the catalytic activity of the Ni_3_S_2_ (101) facet; the details can be found in ref. 26. The Ni_1_–S bond length is 2.21 Å, the Ni_2_–S bond length is 2.32 Å, and the Ni_1_–S–Ni_2_ bond angle is 65.60°. It exhibits a continuous nickel–nickel network structure inside, benefiting charge transport.^17^Fig. 1(b) shows the electronic density of states (DOSs), and the bands across the Fermi level with its metallicity. Near the Fermi level, the DOS is mainly from the Ni-3d and S-3p orbitals. In the OER process, there are four electron transfer steps; each step involves the removal of a proton, as shown in Fig. 1(c). In the first step, H_2_O is adsorbed on the active site Ni atom and loses H^+^, forming HO* with a free energy of 0.96 eV. In the second step, HO* loses another H^+^ to form O* with a free energy of 2.62 eV. In the third step, the second H_2_O is adsorbed and loses another H^+^ proton, forming HOO* with a free energy of 4.06 eV. In the fourth step, the bond is formed between the two oxygen atoms and separates from the surface to form O_2_.^27–30^ Among them, the second step of HO* to O* adsorption is the rate-determining step with the overpotential of 0.43 V, and the binding between the active site Ni and the oxygen-containing intermediate O atoms comes mainly from the interaction Ni-3d_*xz*_ and O-2p_*y*_ orbitals, and Ni-3d_*z*2_ and O-2p_*x*_ orbitals, as shown in inset in Fig. 1(c).

**Fig. 2:**
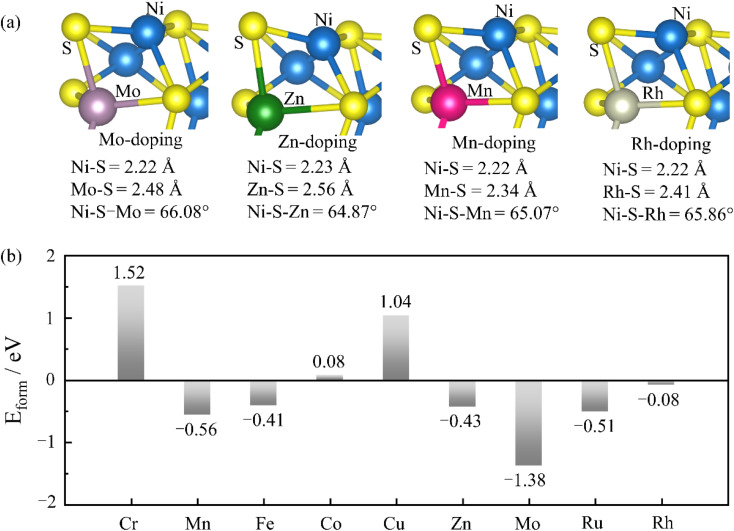
(a) Model diagrams of Ni_3_S_2_ doped with Mo, Zn, Mn, and Rh and (b) the formation energy of the TM-doped system.

Fig. 2(a) shows the typical structure of Mo, Zn, Mn, and Rh-doped systems. TM doping does not break the structure of Ni_3_S_2_ but has a certain influence on the bond length of Ni–S and the bond angle of Ni_1_–S–Ni_2_. We calculated the formation energy to investigate the stability of TM doping. *E*_f_ = *E*_TM@X_ − *E*_X_ − (*μ*_TM_ − *μ*_Ni_), *E*_TM@X_, *E*_X_, *μ*_TM_, and *μ*_Ni_ represent the energy of the TM-doped Ni_3_S_2_ system, pure Ni_3_S_2_ system, and the chemical potential of TM and Ni atoms, respectively. Under O-poor growth conditions, *μ*_TM_ and *μ*_Ni_ are determined by one TM and Ni atom in their respective bulks. The formation energies of Cr, Mn, Fe, Co, Cu, Zn, Mo, Ru, and Rh doping (see Fig. 2(b)) are 1.52, −0.56, −0.41, 0.08, 1.04, −0.43, −1.38, −0.51 and −0.08 eV, respectively. The negative formation energy indicates that doping can be easily achieved in experiments. Among them, Mo-doping has better stability with a formation energy of −1.38 eV, but the stability of Cr, Cu and Co doping is very weak because of their positive formation energies of 1.52, 1.04 and 0.08 eV, respectively.

### 3.2 OER performance of the TM-doped Ni_3_S_2_ (101) facet

**Fig. 3:**
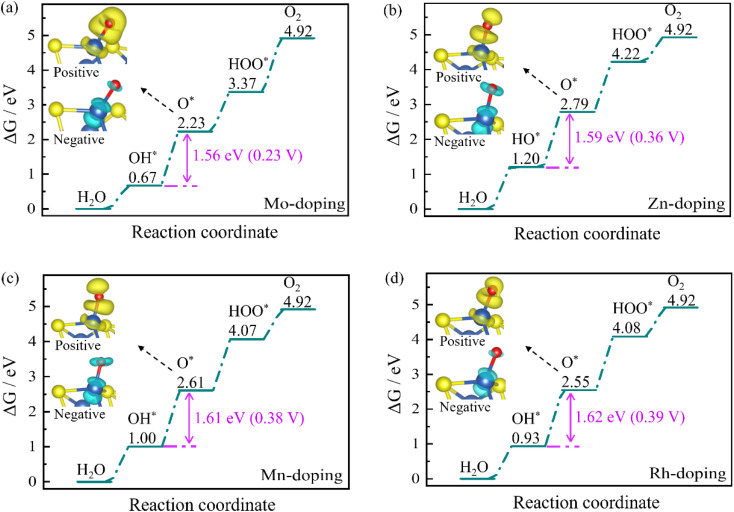
Free energies of (a) Mo, (b) Zn, (c) Mn, and (d) Rh doped Ni_3_S_2_ (101) facets during the OER process. The insets are charge density differences, with charge accumulation and depletion represented by the yellow and blue regions, respectively. The Ni atom acts as the active site.

Fig. 3 shows the free energy step diagrams of TM-doped Ni_3_S_2_ during the OER process. Each step is an uphill endothermic reaction. To make the reaction exothermic, a certain overpotential must be applied during each step of the reaction, allowing for the smooth progress of the reaction. The results indicate that the rate-determining steps of all TM-doping Ni_3_S_2_ remain the second step of HO* to O*, and overpotential decreases to 0.23, 0.36, 0.38 and 0.39 V for Mo, Zn, Mn, and Rh doping, respectively. In particular, the overpotential of Mo-doping is the smallest (0.23 V), which is significantly lower than that of pure Ni_3_S_2_ (0.43 V). Furthermore, we investigate the charge density difference at the rate-determining step (see insets in Fig. 3). The active site Ni atom loses the charges, and the O* atom gains the charges. Typically, the Mo doping exhibits a more pronounced charge transfer (see inset in Fig. 3(a)), greatly improving the interaction between the active site and the adsorbed O* atom.

**Fig. 4:**
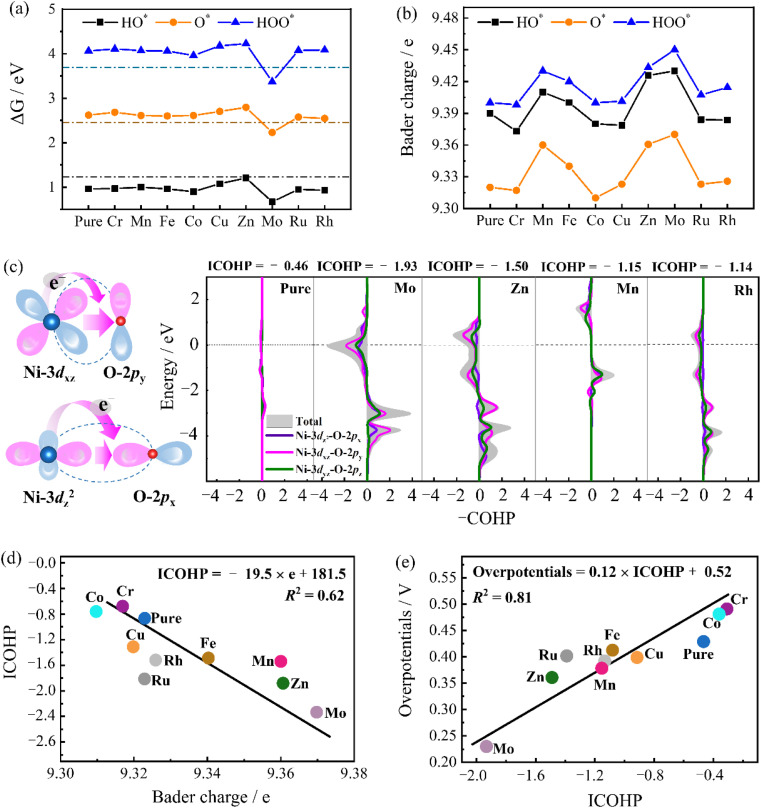
(a) Comparison of the free energy of intermediates in the OER process with the ideal value, (b) Bader charge on the active site Ni atom, (c) the schematic diagram of the orbital coupling and −COHP of Ni–O, (d) the linear dependence between Bader charge and ICOHP, and (e) the linear dependence between the ICOHP and overpotential on pure and TM-doping Ni_3_S_2_ (101) facet at the rate-determining step.

Fig. 4(a) shows the free energies of oxygen-containing intermediates (HO*, O*, HOO*) in different doped systems compared with the ideal value. The free energy of the OER intermediate can be defined as follows:

1 Δ*G*_HO*_ = Δ*G*_1_

2 Δ*G*_O*_ = Δ*G*_1_ + Δ*G*_2_

3 Δ*G*_HOO*_ = Δ*G*_1_ + Δ*G*_2_ + Δ*G*_3_

where Δ*G*_1_, Δ*G*_2_, and Δ*G*_3_ are the free energies of the first, second, and third steps, respectively.^31^ The ideal values of Δ*G*_HO*_, Δ*G*_O*_, and Δ*G*_HOO*_ are 1.23, 2.46, and 3.69 eV, respectively, during the OER (as shown by the dashed horizontal line in Fig. 4(a)). Compared with the ideal values, higher intermediate free energy indicates weaker binding and lower intermediate free energy indicates stronger binding, which is not favorable for desorption.^31^ For the Ni_3_S_2_ (101) facet, the Ni–O interaction shows strong binding at the HO* adsorption but weak binding at the O* and HOO* adsorption. The large energy difference between the HO* and O* adsorption demonstrates that the second step is the rate-determining step. Taking Mo-doping as an example, the rate-determining step Δ*G*_O*_ is 2.23 eV, and the pure Ni_3_S_2_ (101) facet Δ*G*_O*_ is 2.62 eV, which is reduced by 0.39 eV, resulting in lower overpotential in the OER process. In an experiment, the catalysts may be oxidized and covered with HO* or O*, and form the metal oxyhydroxide or the metal oxide, which affects the OER efficiency. However, these require specific conditions, such as high current density and specific pH conditions.^32^ In the OER process, the Ni_3_S_2_ shows good stability and exhibits superior OER activity during the OER process, and significant surface oxidation cannot be found.^33^ By the comparison between the free energies of oxygen-containing intermediates (HO*, O*, HOO*) and the ideal values, as shown in Fig. 4(a), we can observe that the TM doping effectively improves the binding between the surface and intermediates, benefiting the intermediate adsorption and desorption. This indicates that TM doping improves structural reconstruction to some extent.

The Bader charge distribution on the active site Ni atom is shown in Fig. 4(b). More charges are concentrated on the active site Ni atom at each electron-transfer step for TM doping, except for Cr and Co doping due to its structural instability. The increased charge distribution of the Ni atom makes it exhibit higher catalytic activity, thereby enhancing the binding between the Ni–O orbitals. Here, we propose a mechanism for TM doping that modulates p–d orbital coupling in the OER process (Fig. 4(c)). TM doping increases the charge distribution on the active site, donating more charges to the adsorbed O atom and enhancing the orbital hybridized coupling between Ni-3d and O-2p. Therefore, the conversion of HO* to O* becomes easier in the rate-determining step, and the overpotential is reduced. The binding between active site Ni and O* can be found in the negative Crystal Orbital Hamilton Population (−COHP) (Fig. 4(c)), which mainly comes from Ni-3d_*xz*_ and O-2p_*y*_, and Ni-3d_*z*2_ and O-2p_*x*_ orbitals. The integrated values (ICOHP) can quantitatively describe the interaction between the surface Ni atom and the oxygen-containing intermediates.^34–37^ The more negative ICOHP indicates, the stronger the interaction between the Ni and O atoms. The ICOHP in the pure Ni_3_S_2_ (101) facet is −0.46. Mo, Zn, Mn and Rh doping decreases to −1.93, −1.50, −1.15, and −1.14, respectively. The introduction of TM atoms reduces the ICOHP and enhances the orbital coupling of Ni-3d_*xz*_ and O-2p_*y*_, and Ni-3d_*z*2_ and O-2p_*x*_ orbitals.

Fig. 4(d) and (e) show the linear dependencies between the Bader charge and ICOHP, and ICOHP and overpotential in the rate-determining step, which can be fitted as follows:ICOHP = −19.5 × *e* + 181.5** **(*R*^2^ = 0.62)Overpotentials = 0.12 × ICOHP + 0.52** **(*R*^2^ = 0.81)where *e* is the Bader charge and *R*^2^ is the linear correlation. The closer *R*^2^ is to 1, the higher the linear correlation between the independent and dependent variables.^38,39^ Here, the ICOHP becomes more negative as the Bader charge increases. The overpotential decreases as the negative value of the ICOHP increases. Thus, it can be observed that TM doping increases the Bader charge of the active site, enhancing the Ni-3d and O-2p orbital coupling and reducing the overpotential of OER. Among them, Mo doping achieves a greater Bader charge on the active site and lowers ICOHP. Therefore, it has lower overpotential and greatly enhances OER performance.

## 4. Conclusion

In this paper, the DFT method was used to investigate how TM doping modulates the orbital coupling between Ni-3d and O-2p states to improve the OER performance of the Ni_3_S_2_ (101) facet. The results show the following: (1) TM doping induces charge redistribution and enhances interaction between the active site and the oxygen-containing intermediates, which mainly comes from the strong orbital interaction of Ni-3d_*xz*_ and O-2p_*y*_, and Ni-3d_*z*2_ and O-2p_*x*_ orbitals. (2) The interaction between the Ni and O atoms is linearly dependent on the overpotential, and the overpotential decreases with an increase in the bonding interaction. (3) Mo doping shows the optimal adjustment effect, and it has a strong interaction between Ni–O at the rate-determining step and a lower overpotential of 0.23 V. The TM-doping Ni_3_S_2_ (101) facet may be a feasible strategy for improving the OER performance and providing theoretical references for experiments.

## Conflicts of interest

There are no conflicts to declare.

## Supplementary Material

NA-006-D4NA00503A-s001

## Data Availability

Additional data are made available in the ESI† of this manuscript.

## References

[cit1] Guo X., Duan M., Zhang J., Xi B., Li M., Yin R., Zheng X., Liu Y., Cao F., An X., Xiong S. (2022). Adv. Funct. Mater..

[cit2] Zhang H., Wan F., Li X., Chen X., Xiong S., Xi B. (2023). Adv. Funct. Mater..

[cit3] Guo X., Shi J., Li M., Zhang J., Zheng X., Liu Y., Xi B., An X., Duan Z., Fan Q., Gao F., Xiong S. (2023). Angew Chem. Int. Ed. Engl..

[cit4] Guo X., Zhang J., Yuan L., Xi B., Gao F., Zheng X., Pan R., Guo L., An X., Fan T., Xiong S. (2023). Adv. Energy Mater..

[cit5] Li Y., Feng A., Dai L., Xi B., An X., Xiong S., An C. (2024). Adv. Funct. Mater..

[cit6] Li P., Zhao S., Huang Y., Huang Q., Xi B., An X., Xiong S. (2024). Adv. Energy Mater..

[cit7] Liang Q., Brocks G., Bieberle-Hütter A. (2021). J. Phys.: Energy.

[cit8] Jamesh M.-I., Harb M. (2021). J. Energy Chem..

[cit9] Chen C., Sun M., Zhang F., Li H., Sun M., Fang P., Song T., Chen W., Dong J., Rosen B., Chen P., Huang B., Li Y. (2023). Energy Environ. Sci..

[cit10] Chen L., Wang Y., Zhao X., Wang Y., Li Q., Wang Q., Tang Y., Lei Y. (2022). J. Mater. Sci. Technol..

[cit11] Fang Z., Tang Z., Lin S., Li R., Chen X., Tian J., Liu L., Peng J., Liu S., Fu B., Deng T., Wu J. (2023). CrystEngComm.

[cit12] Li W., Liu R., Yu G., Chen X., Yan S., Ren S., Chen J., Chen W., Wang C., Lu X. (2023). Small.

[cit13] Chen S., Huang H., Jiang P., Yang K., Diao J., Gong S., Liu S., Huang M., Wang H., Chen Q. (2020). ACS Catal..

[cit14] Lee H., Kim J. Y., Lee S. Y., Hong J. A., Kim N., Baik J., Hwang Y. J. (2018). Sci. Rep..

[cit15] Zhang J., Wang T., Pohl D., Rellinghaus B., Dong R., Liu S., Zhuang X., Feng X. (2016). Angew. Chem..

[cit16] Chung D. Y., Han J. W., Lim D. H., Jo J. H., Yoo S. J., Lee H., Sung Y. E. (2015). Nanoscale.

[cit17] Dong J., Zhang F.-Q., Yang Y., Zhang Y.-B., He H., Huang X., Fan X., Zhang X.-M. (2019). Appl. Catal., B.

[cit18] Shao Z., Liu R., Xue H., Sun J., Guo N., He F., Wang Q. (2021). Chem. Eng. J..

[cit19] Yuan C. Z., Sun Z. T., Jiang Y. F., Yang Z. K., Jiang N., Zhao Z. W., Qazi U. Y., Zhang W. H., Xu A. W. (2017). Small.

[cit20] Kresse G., Hafner J. (1994). Phys. Rev. B: Condens. Matter Mater. Phys..

[cit21] Perdew J. P., Burke K., Ernzerhof M. (1996). Phys. Rev. Lett..

[cit22] Blochl P. E. (1994). Phys. Rev. B: Condens. Matter Mater. Phys..

[cit23] Kresse G., Furthmüller J. (1996). Comput. Mater. Sci..

[cit24] Dong G., Liu J., Xu X., Pan J., Hu J. (2023). J. Electroanal. Chem..

[cit25] Zhang M., Shao X., Liu L., Xu X., Pan J., Hu J. (2022). RSC Adv..

[cit26] Shuang W., Huang H., Kong L., Zhong M., Li A., Wang D., Xu Y., Bu X.-H. (2019). Nano Energy.

[cit27] Mohammed-Ibrahim J. (2020). J. Power Sources.

[cit28] Li Q., Kang Z., Guo L., Hu J., Pan J. (2023). J. Electroanal. Chem..

[cit29] Wang Z., You J., Zhao Y., Yao R., Liu G., Lu J., Zhao S. (2023). J. Environ. Chem. Eng..

[cit30] Li Z., Xu X., Lu X., He C., Huang J., Sun W., Tian L. (2022). J. Colloid Interface Sci..

[cit31] Liang Q., Brocks G., Sinha V., Bieberle-Hütter A. (2021). ChemSusChem.

[cit32] Liu J., Qiao W., Zhu Z., Hu J., Xu X. (2022). Small.

[cit33] Li B., Li Z., Pang Q., Zhang J. Z. (2020). Chem. Eng. J..

[cit34] Niu H., Wang X., Shao C., Zhang Z., Guo Y. (2020). ACS Sustain. Chem. Eng..

[cit35] Deringer V. L., Tchougreeff A. L., Dronskowski R. (2011). J. Phys. Chem. A.

[cit36] Ndassa I. M., Fokwa B. P. T. (2014). Comput. Mater. Sci..

[cit37] Ren M., Guo X., Huang S. (2021). Appl. Surf. Sci..

[cit38] Liu S., Li Z., Wang C., Tao W., Huang M., Zuo M., Yang Y., Yang K., Zhang L., Chen S., Xu P., Chen Q. (2020). Nat. Commun..

[cit39] Liu J., Lv X., Ma Y., Smith S. C., Gu Y., Kou L. (2023). ACS Nano.

