# Supplementary material for: TM-doping modulated p–d orbital coupling to enhance the oxygen evolution performance of Ni3S2
Source: Nanoscale Adv. 2024 Aug 16;6(20):5158–63. doi: 10.1039/d4na00503a (PMC11376051; doi:10.1039/d4na00503a)
Supplement: NA-006-D4NA00503A-s001 [file NA-006-D4NA00503A-s001.pdf]

Supplementary Information (SI) for Nanoscale Advances.
This journal is © The Royal Society of Chemistry 2024

Supporting Information

TM-doping Modulated *p-d* Orbital Coupling to Enhance the Oxygen Evolution Performance of Ni_3S_2

1. The influence of the relativistic and dispersion effects on the OER process.

Here, we consider the relativistic and dispersion effects. Taking the pure, Mo-doped and Mn-doped systems as examples, the formation energy and the free energy of the OER process are discussed, respectively. When the relativistic and dispersion effects are not considered, the formation energies of Mo and Mn doping are −1.38 and −0.56 eV, respectively. When the relativistic and dispersion effect are considered, the formation energies of Mo and Mn doping are −0.81 and −0.24 eV, respectively, which is lower than that of without dispersion effects, but both exhibit the similar trend. The diagram of the free energy steps for the OER process is shown in Fig. S1. After considering the relativistic and dispersion effect, the general trend of the reaction remained unchanged, and the overpotential of the pure, Mo and Mn doping systems changed from 0.43, 0.23 and 0.38 V to 0.41, 0.22 and 0.36 V, the results are almost the same. As a result, the relativistic and dispersion effects have little effect on these doped systems.

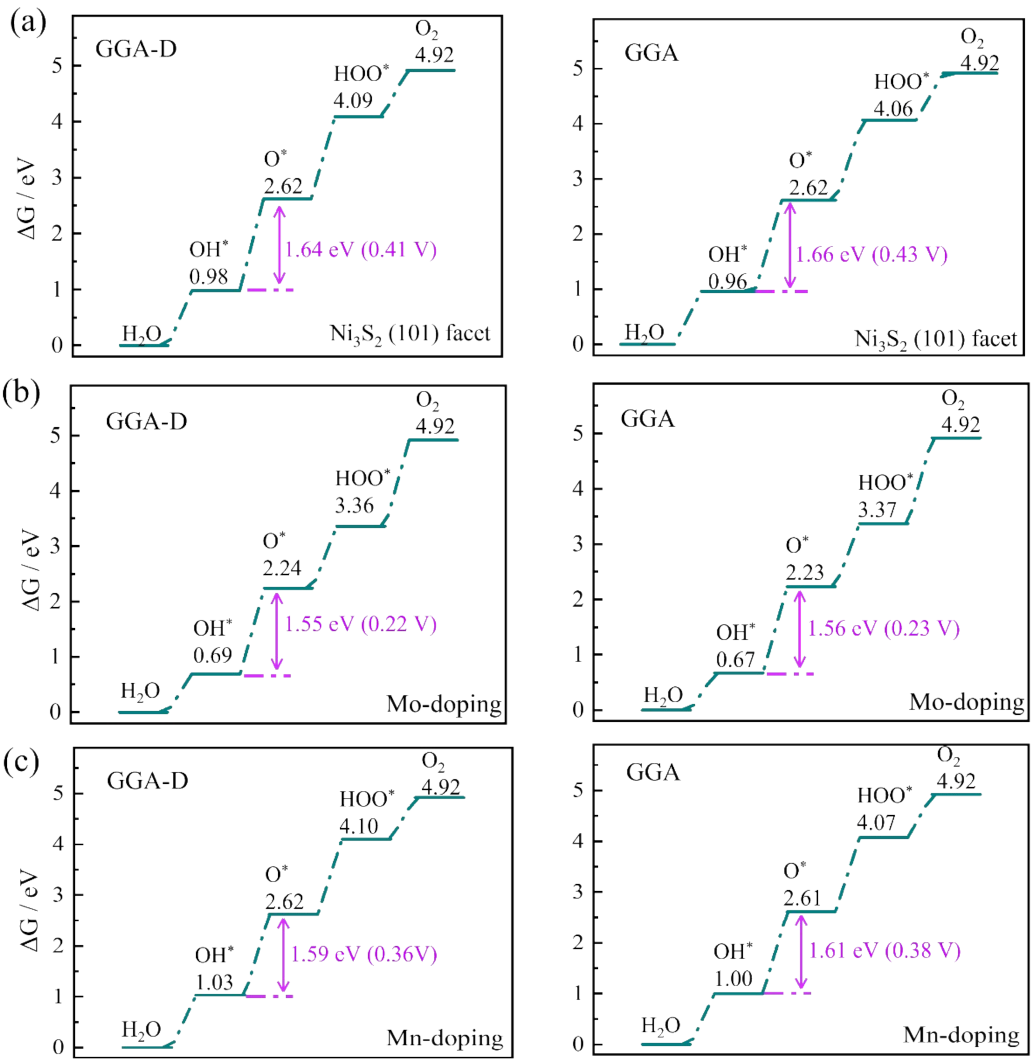

Fig. S1 The free energies of (a) pure, (b) Mo, (c) Mn doped Ni_3S_2 during the OER process under dispersion effect and without dispersion effect.

2. The calculation details of the free energy for the OER process.

The free energy of the OER process is defined by the equation as: $\Delta G = \Delta E + \Delta \mathrm{ZPE} - T\Delta S$, the details are as following:

$$\Delta G_1 = E\left(\mathrm{HO}^*\right) - E_{\mathrm{surf}} - E_{\mathrm{H_2O}} + \frac{1}{2}E_{\mathrm{H_2}} + (\Delta \mathrm{ZPE} - T\Delta S) - eU \tag{1}$$

$$\Delta G_2 = E\left(\mathrm{O}^*\right) - E\left(\mathrm{HO}^*\right) + \frac{1}{2}E_{\mathrm{H_2}} + (\Delta \mathrm{ZPE} - T\Delta S) - eU \tag{2}$$

$$\Delta G_3 = E\left(\mathrm{HOO}^*\right) - E\left(\mathrm{O}^*\right) - E_{\mathrm{H_2O}} + \frac{1}{2}E_{\mathrm{H_2}} + (\Delta \mathrm{ZPE} - T\Delta S) - eU \tag{3}$$

$$\Delta G_4 = E_{\text{surf}} - E\left(\text{HOO}^*\right) + E_{\text{O}_2} + \frac{1}{2}E_{\text{H}_2} + (\Delta\text{ZPE} - T\Delta S) - eU \tag{4}$$

where E refers to the reaction energy of each electron-transfer step, ΔZPE and ΔS represent zero-point energy and entropy contribution of the geometry, which are obtained by calculating the vibrational frequencies of the reactants and products in the gas phase using standard tables. The calculated vibrational frequencies of the OH^*, O^*, and HOO^* species can be found in the following Table R1. The total reaction $H_2O \rightarrow 1/2O_2 + H_2$ requires a voltage of 2.46 V, the minimum free energy should cause the two water molecules to split at a voltage of 4.92 V, the rate-determining step is the reaction step corresponding to the highest free energy change (ΔG_{max}), and the overpotential of the determining step U_{pds} is ($\Delta G_{max}-1.23$) V. We have added the calculation details in the supporting information.

Table R1 the energy values of the oxygen-containing intermediates involved in the calculation, and the zero-point energy (ZPE) and entropy correction (TS) of the adsorbed HO^*, O^* and HOO^*, T=298.15 K, unit is eV.

	Pure Ni_3S_2	Cr doping	Mn doping	Fe doping	Co doping	Cu doping	Zn doping	Mo doping	Ru doping	Rh doping
E_{surf}	-154.80	-158.63	-158.31	-157.23	-156.22	-152.61	-150.02	-159.22	-157.70	-156.25
E_{HO*}	-164.96	-168.77	-168.45	-167.43	-166.47	-162.68	-159.98	-169.66	-167.89	-166.47
E_{O*}	-159.64	-163.39	-163.09	-162.08	-161.05	-157.36	-154.68	-164.40	-162.57	-161.15
E_{HOO*}	-169.34	-173.14	-172.83	-171.77	-170.84	-167.04	-164.38	-174.44	-172.25	-170.78
$(ZPE\text{-}TS)_{HO*}$	0.30	0.29	0.32	0.35	0.33	0.32	0.34	0.29	0.32	0.32
$(ZPE\text{-}TS)_{O*}$	0.03	0.03	0	0.02	0.02	0.03	0.03	0	0.03	0.02
$(ZPE\text{-}TS)_{HOO*}$	0.37	0.38	0.35	0.35	0.33	0.37	0.34	0.35	0.38	0.37
